# Distraction osteogenesis versus induced membrane technique for infected tibial non-unions with segmental bone loss: a systematic review of the literature and meta-analysis of available studies

**DOI:** 10.1007/s00068-023-02375-w

**Published:** 2023-11-03

**Authors:** Sophia M. Wakefield, Costas Papakostidis, Vasileios P. Giannoudis, Alfonso Mandía-Martínez, Peter V. Giannoudis

**Affiliations:** 1 Academic Department of Trauma and Orthopaedics Surgery, School of Medicine, University of Leeds, Leeds General Infirmary, Clarendon Wing, Floor D, Great George Street, Leeds, LS1 3EX UK; 2 NIHR Leeds Biomedical Research Centre, Chapel Allerton Hospital, Leeds, UK

**Keywords:** Tibia, Non-union, Infection, Distraction osteogenesis, Induced membrane technique, Masquelet, Bone defect

## Abstract

**Introduction:**

Infected tibial non-unions with associated bone loss can be challenging to manage. At present, the two main methods utilized in the management of these fractures include the Ilizarov technique of Distraction Osteogenesis (DO) using external fixator devices, or alternatively, the Induced Membrane Technique (IMT), devised by Masquelet. As there is a paucity of data directly comparing the outcomes of these techniques, there is no universal agreement on which strategy a surgeon should choose to use.

**Aims:**

This systematic review and meta-analysis aimed to summarize the outcomes of both DO and IMT, in terms of primary outcomes (bone union and infection elimination), and secondary outcomes (complication rates and functional outcomes).

**Methods:**

A PRISMA strategy was used. Medline, Web of Science, Cochrane Central Register of Controlled Trials (CENTRAL), and Google Scholar library databases were interrogated using pre-defined MeSH terms and Boolean operators. Quality of evidence was evaluated using OCEBM and GRADE systems.

**Results:**

Thirty-two studies with 1136 subjects met the inclusion criteria. With respect to the primary outcomes of interest, union was observed in 94.6% (DO method) and 88.0% (IMT method); this difference, however, was not significant between the two techniques (*p = *0.45). In addition, infection elimination rates were also higher in the Ilizarov DO group when compared to Masquelet (Mq) IMT (93.0% vs 80.4% respectively). Again, no significant difference was observed (*p = *0.06). For all secondary outcomes assessed (unplanned re-operations, re-fracture rates amputation rate), no statistically significant differences were documented between the treatment options.

**Conclusion:**

This study demonstrated that there is no clinical difference in outcomes for patients treated with Ilizarov DO versus Mq IMT techniques. The evidence base at present is relatively sparse and, therefore, we would recommend for further Level I studies to be conducted, to make more meaningful conclusions.

## Introduction

Infected tibial non-unions (NUs), particularly when associated with segmental bone loss, pose a great therapeutic challenge to the treating surgeon. The precarious soft tissue envelope, often combined with soft tissue loss and exposed bone, further compound treatment efforts for infection eradication and bone healing. Other critical issues to be addressed include intercalary bone loss (either due to the initial injury or subsequent surgical debridement); deformities; leg-length discrepancies (LLDs); failed metalwork; and polymicrobial infections with resistant microorganisms [1, 2]. The fundamental targets of treatment are infection elimination, osseous reconstruction, and bony union. Various techniques have evolved to serve these purposes [3].

The distraction osteogenesis (DO) technique, originally developed by Professor Gavriil Ilizarov in the 1950s [4], is founded on the “law of tension stress”, in which controlled and slow traction stimulates and promotes tissue regeneration, similar to that of embryonic tissue [5]. The great advantage of this technique lies in that is it can achieve complete infection eradication by completely removing all of the avascular and potentially infected osseous segment. The regenerate bone, formed through bone transport, fills the intercalary osseous gap, whilst LLDs and axial deformities are simultaneously corrected [6, 7]. Union rates as high as 97% have been reported even for large osseous defects of more than 3 cm [8]. Despite the aforementioned advantages, the technique is fraud with high complication rates, such as pin-track infections, axial deviation, adjacent joint stiffness, and wire loosening [9, 10].

The more recently introduced induced membrane technique (IMT) by Professor AC Masquelet was also designed to address large bone defects, regardless of infection status [11]. The process entails at least two stages, the first of which consists of a thorough debridement and removal of all avascular osseous tissue down to bleeding bone, skeletal stabilisation (with either external/internal fixation methods) and a temporary filling of the resulted bone defect with an antibiotic-loaded polymethylmethacrylate** (**PMMA) spacer. This cement spacer is required to induce the formation of a biologically active membrane (hence, the term “IMT”), hosting osteo-inductive growth factors for bone healing [12]. In addition, it is mandatory to address any existing soft tissue defect with appropriate plastic-surgery techniques, as well as eradicating infection and producing a sterile environment for bone grafting at a later stage. This step may need repeating for successful infection elimination [13]. At the second stage (4–6 weeks later), the cement spacer is replaced by autologous cancellous bone graft (occasionally mixed with bone graft alternatives, such as cancellous allografts or gelatin sponge); the addition of these have expanded the indications for this technique in major bone defect management [11, 14–16]. Variable results have been reported in the literature, with the Masquelet (Mq) technique used in the treatment of infected tibial defects of up to 28 cm. The overall success rate (union plus infection elimination) ranged from 45 to 100%, with the unplanned reoperation rate between 2 and 58% [17–20].

Both Ilizarov DO and Mq IMT have proved their merits in the management of large osseous defects (≥ 3 cm). However, a comparison of their outcomes in the adverse environment of septic tibial NUs with concomitant bone loss has not been well-explored. The aim of our systematic review and meta-analysis is to comparatively summarize the outcomes of both techniques in the management of infected tibial NU with segmental bone loss. Our primary outcomes of interest were union rate, and infection elimination rate. The secondary outcome measures focused on complications and functional outcomes related to each method.

## Methods

This study was conducted in accordance with the latest guidelines of the Preferred Reporting Items for Systematic Reviews and Meta-Analyses (PRISMA) statement [21]. Our review was prospectively registered with the PROSPERO database under the following registration number: CRD42023403534.

### Eligibility criteria and literature search

Eligibility criteria were defined prior to a comprehensive search of the relevant literature and were formulated according to the “Population, Intervention, Comparison, Outcome, Study Design” (PICOS) format. Studies were considered eligible if they met the following inclusion criteria. *Population*: Adult cohorts suffering from infected tibial NUs with concomitant bone loss that have been reported in studies published from 2000 to the present date; *Intervention*: Any mode of Ilizarov DO or Mq IMT; *Comparison*: Patient cohorts treated with DO or IMT; *Outcome*: The primary outcomes of interest were bone healing and infection elimination rates. Secondary outcomes of interest considered were complications such as pin-track infection, residual deformity, adjacent joint contracture, re-fracture, as well as unplanned reoperations, and functional outcomes.

To ensure consistency of the results across the component studies, all outcome rates within each primary study were calculated as number of patients with the particular outcome (e.g., NU healing, infection elimination, pin-track infection, etc.) divided by the cohort size of the study; *Study design*: Both experimental (randomised control trials -RCTs) and observational study design (prospective or retrospective cohort studies) were deemed eligible for inclusion.

In the event of a lack of eligible studies directly comparing the two aforementioned techniques, we planned to perform an appropriate proportion meta-analysis for all available outcomes of interest within each treatment group (Ilizarov DO, Mq IMT), with the potential of a subsequent indirect comparison of their outcomes.

Exclusion criteria were: (1) studies with inadequate reporting on at least the primary outcomes of interest; (2) reports with cohort size of less than 10 patients; (3) studies including exclusively paediatric populations; (4) reports on infected NUs of long bones other than the tibia; (5) studies reporting on aseptic NUs; (6) studies including septic tibial NUs without intercalary bone defect; (7) case-reports, experimental or biomechanical studies.

### Search strategy

An electronic search of the Medline database via the PubMed search engine was initially conducted by three independent researchers (SMW, CP, AMM) using the following Medical Subject Headings (MeSH) terms and Boolean operators: (infected OR septic) AND (tibia) AND (NU OR pseudarthrosis). The search was further extended to the Web of Science, Cochrane Central Register of Controlled Trials (CENTRAL), and Google Scholar databases. In addition, the references of both eligible articles and other relevant review articles were manually searched to isolate articles that had been potentially missed by the initial search. No language restrictions were imposed. Titles of journals, names of authors, and institutions were not masked, to avoid duplication of data. The reviewers independently assessed the titles and abstracts of all retrieved articles, and for potentially eligible articles, the full text was obtained and screened against the eligibility criteria. Any disagreements between the reviewers were resolved by discussion in the presence of the senior author (PVG). The search was conducted in March 2023 and was limited to the time period since 1st January 2000 onwards.

### Data extraction

The following data were extracted from each eligible paper and tabulated into a predefined Microsoft Excel spreadsheet: demographic data and baseline characteristics; sample size; data source; enrolment period; type of procedure (Ilizarov or Mq technique); number of bone unions; number of infection eradications; categorisation of the ultimate functional results; and type and number of complications.

### Assessment of the risk of bias

Initially, each primary study was assessed based on its OCEBM Level of Evidence (LoE) [22]. The assessment of the risk of bias (ROB) of RCTs was based on the revised Cochrane Risk of Bias tool for randomised trials (RoB2) [23]. RoB2 is structured into the following bias domains: (i) randomisation process; (ii) deviations from intended interventions; (iii) missing outcome data; (iv) measurement of the outcome; (v) selection of the reported result. The overall risk of bias generally corresponds to the worst risk of bias in any of the domains. For non-randomised primary studies, the Methodological Index for Non-Randomised Studies (MINORS) critical appraisal tool was used to evaluate the potential risk of bias of their evidence [24]. The MINORS tool, which is specifically designed to assess the methodological quality in surgical intervention studies, is composed of 8 methodological items applicable to all non-randomised studies, plus 4 additional items for non-randomised comparative studies. As each item receives a maximum score of 2 points, the ideal global score for non-comparative and comparative studies is 16 and 24 points, respectively.

Each main outcome of interest was assessed in terms of quality of evidence based on the adjusted Grading of Recommendations Assessment, Development and Evaluation (GRADE) framework [25]. The GRADE framework provides 7 factors, with each one being rated as either “no serious limitations” or “serious limitations”. A risk factor that had 5 or more scores of “no serious limitations” were considered high quality. Those with 3 or 4 scores of “no serious limitations” were considered “moderate quality”, while risk factors with less than 3 scores of “no serious limitations” were deemed “low quality”.

### Statistical analysis

For studies with single intervention cohorts (either Ilizarov or Mq treatment cohort), all outcomes of interest were expressed as proportions (e.g., union rate or infection-elimination rate). Pooling of proportions was done with the MedCalc® Statistical Software version 20.114 (MedCalc Software Ltd, Ostend, Belgium; https://www.medcalc.org; 2022) using a random effects model (DerSimonian and Laird), as we assumed that the cohorts within the primary studies were not identical and, therefore, the true effect size was not the same across those studies. Statistical heterogeneity was detected with the use of Cochran’s *Q* test and Higgins *I*^2^ test [26, 27]. The level of statistical significance was set at 0.1 for the *Q* test, as it is characterised by low sensitivity for detecting heterogeneity. The *I*^2^ test is bound at its upper end by 100% and values up to 25%, between 25 and 50% and over 50% were considered to represent a low, modest and high degree of heterogeneity, respectively. Only in the complete absence of statistical heterogeneity (*I*^2^ close to 0%) would a fixed effects model be used. The results of pooling were illustrated as forest plots. The Mann–Whitney *U* test was used for the non-parametric comparisons of numerical data between different treatment groups.

For studies with comparator cohorts, binary outcomes of interest were expressed as risk ratios (RRs), with respective 95% confidence intervals (95% CIs). Pooling of data was done with the RevMan (5.3) software (Review Manager, Nordic Cochrane Centre, Copenhagen, Denmark) using the Inverse Variance statistical method and a fixed or random effects model, based on our previous assumptions.

### Publication bias

The potential presence of publication bias was firstly explored visually by generating the respective funnel plots for the main outcomes of interest. A symmetrical distribution of the studies about the pooled effect estimate would be interpreted as an absence of publication bias. Furthermore, we utilised the Egger’s test and the Begg’s rank test [28, 29]. For both tests, there is an indication of publication bias when the two-sided *p*-value is very low (below the significance level).

### Subgroup analysis

To explore the effect of the potential presence of heterogeneity on the final outcomes, certain subgroups of the initial cohort were determined with the outcomes of interest, and were calculated within each subgroup. Subgroups were identified based on the type of the external fixation device (traditional circular frames or modern unilateral external fixator) for the DO method, whereas, for the Mq IMT technique, on the type of stabilisation of the NU (external or internal fixation methods).

### Sensitivity analysis

Sensitivity analysis was performed by repeating the pooling process after eliminating studies of either low methodological rating by the MINORS tool, or dubious eligibility. Should this process not yield considerably different results than the originally obtained, our confidence surrounding the robustness of our findings would increase.

## Results

**Fig. 1:**
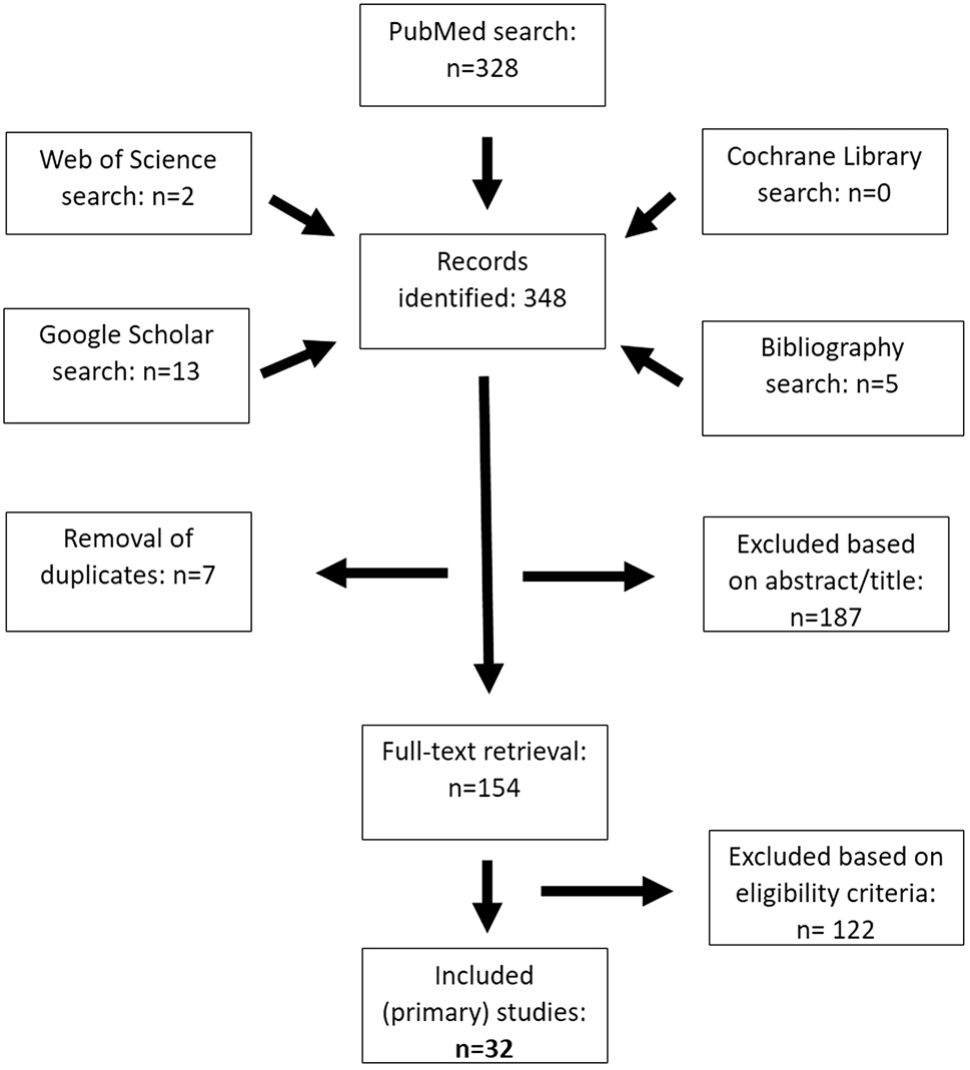
Preferred reporting items for systematic reviews and meta-analyses (PRISMA) flowchart

The initial PubMed search yielded 328 citations. Fifteen additional records were identified through the electronic search of other electronic databases, while 5 records were found through a manual search of relevant bibliographies. After duplicates were removed, 341 titles and abstracts were screened for suitability. Most studies (*n = *187) were excluded based on the information provided in the title and abstract. One-hundred and fifty-four (*n = *154) articles were ultimately retrieved for full-text review. After applying the eligibility criteria, 122 articles were excluded, leaving a final 32 primary studies [17–20, 30–57] for analysis, as summarised in the PRISMA flowchart (Fig. 1).

There were only two comparative studies of Mq against the Ilizarov technique [39, 54]. In addition, one report comprised of tibial and femoral infected NUs. The only available data pertaining to tibial NUs were used in the pooled analysis [39]. The remaining articles included single-intervention cohorts of either the Ilizarov method (*n = *22) [30–38, 40, 42–51, 53, 56] or the Mq technique (*n = *8) [17–20, 41, 52, 55, 57].

All 32 primary studies comprised of a total population of 1136 patients. The primary studies with single-intervention cohorts included 1085 patients, of whom 865 patients had been treated with the Ilizarov method and the remaining 215 with the Mq technique. The two comparative studies comprised of 51 patients [39, 54]. Twenty-six patients had been treated with DO, while the remaining 25 with the Mq IMT technique. The baseline and demographic characteristics, follow-up details, and sources of clinical diversity of all primary studies are depicted in Tables 1 and 2.

**Table 1:** Baseline and demographic characteristics

Sort	Authors	Published year	Treatment period	Study type	*n*-pts	Age: mean (range), years	M:F	Bone gap (cm): mean (range)	Treat-ment method	Type of fixation
1	Magadum [30]	2006	1997–2000	R	27	39 (15–65)	27:0	10 (6–17)	DO (ACRL)	CF
2	Baruah [31]	2007	1994–2003	RC (TFW vs Hybrid)	50	(18–56)	45:5	3.5 (2–9)	DO	CF
3	Madhusudhan [32]	2008	nd	P	22	37.2 (20–52)	18:4	(2–9)	DO	CF
4	Bumbasirevic [33]	2010	1991–1996	R	30	30.4 (20–49)	29:1	6.9 (4–11)	DO	CF
5	Liu [34]	2012	2000–2008	R	35	37.3 (18–64)	25:10	7.9 (4–10.5)	DO	MF (LRS)
6	Mora [35]	2014	1986–2010	R	120	34 (21–57)	92:28	9.5 (6–18)	DO	CF
7	Xu [36]	2014	2003–2011	R	30	34.1 (19–49)	21:9	6.4 (3–12)	DO	CF
8	Moghaddam [17]	2015	2010–2014	RC (Mq vs 1-stage BG)	50	47.8 (15–72)	42:8	4 (1–26)	Mq	Pl: 26, IMN: 22, EF: 1, none: 1
9	Barakat [37]	2017	nr	P	30	40 (18–67)	27:3	3.85 (2.5–6)	DO (ASRL)	CF
10	Meleppuram [38]	2017	2012–2015	R	42	38 (26–64)	32:10	(2.5–5.5)	DO	CF
11	Tong [39]	2017	2012–2015	RC (Mq/DO)	Mq: 13, DO: 13	Mq: 23 ± 9.5 DO: 27.5 ± 10	nr	Mq: 6.7 ± 3.7, DO: 6.8 ± 3.4	Mq vs DO	Mq: Pl., MF, CF DO: MF, CF
12	Morris [18]	2017	2010–2015	R	12	35 (16–62)	9:3	5.8 (2–15)	Mq	Pl: 2, IMN: 8, CF: 1
13	Tetsworth [40]	2017	nr	RC (BT/ASRL)	42	38.7 (18–76)	36:6	6.4 (3–10)	DO	CF
14	Luo [57]	2017	2012—2015	R	67	32 (16–61)	58:9	6.75 (2–16)	Mq	IMN
15	Siboni [41]	2018	2007–2014	R	18 (19 cases)	53.9 [± 16.7]	14:4	5.2 (1.1–18)	Mq	Pl: 8, EF: 7, none: 4
16	Bakhsh [42]	2019	2015–2017	R	56	32.6 (16–50)	53:3	4.3 (3–9)	DO	CF
17	Rohilla [43]	2019	2011–2016	RCT (MF vs CF)	30	32.54 (18–60)	29:1	5.25 (3–7)	DO	MF: 15, CF: 15
18	Jilani [44]	2019	2014–2016	P	22	31 (16–55)	17:5	4.7 (2–9)	DO	MF (LRS)
19	Sharma [45]	2019	2016–2019	P	30	32.5 (18–51)	23:7	4.85 (2.15–8.5)	DO	CF
20	Kinik [51]	2019	2000–2017	R	30	39.5 (16–68)	28:2	8.1 (6–15)	DO	CF
21	Baruah [46]	2020	1997–2010	RC (ASRL/BT)	86	37.3 (18–65)	64:22	4 (3–9)	DO	CF
22	Sigmund [47]	2020	nr	P (ASRL/BT)	47	50 (21.78)	nr	5.1 (2–10)	DO	CF
23	Kushwaha [48]	2020	2016–2017	P	21	29.4 (7–61)	19:2	2.3 (1.5–4)	DO	MF (LRS)
24	Wadhwani [49]	2020	2013–2016	RC	35	29.6 (18–65)	30:5	5.9 (2–10)	DO	MF (LRS)
25	Yushan M [50]	2020	2006–2016	RC	37	40 (18–57)	28:9	8.7 (5–13.5)	DO	MF (LRS)
26	Pesciallo [19]	2021	2012–2019	R	13	40 (18–68)	10:3	4.2 (4–6)	Mq	IMN: 11 Plate: 2, EF: 1
27	Lotzien [52]	2021	2012–2017	R	31	45.8 (18–77)	30:1	8.3 (1.7–27.8)	Mq	CF
28	Gupta [53]	2021	2018–2020	P	18	32.3 (19–55)	16:2	3.1 (2–5)	DO	MF (LRS)
29	Rohilla [54]	2021	2016–2018	RCT (BTvs Mq)	BT: 13, MQ: 12	31.7 (18–60), 39.7 (25–60)	12:1, 11:1	3.9 (3–6), 3.8 (2–6)	BTMq	CF: 4, MF: 9, CF: 3, MF: 9
30	Yang [20]	2022	2017–2020	R	12	44.5 (19–65)	11:1	2.67 (1–6)	Mq	CF: 12
31	Ozpolat [55]	2022	2016–2019	R	11	40.4 (25–63)	11:0	5.1 (2.5–9.8)	Mq	Pl.: 2, IMN: 9
32	Corona [56]	2022	2010–2018	R	31	41 (18–72)	27:4	9.1 (5.2–15.3)	DO (BT: 15, AS: 11, ASRL: 3)	CF: 17, MF: 12

Hybrid (assembly): combination of transfixing wires with pins in circular frames. LRS: (The Orthofix) Limb Reconstruction System

*AS* acute shortening, *ASRL* acute shortening re-lengthening, *BG* bone grafting, *BT* bone transport, *CF* circular frames, *DO* distraction osteogenesis, *EF* external fixation, *MF* monolateral frame, *Mq* masquelet (technique), *nr* not reported, *P* prospective, *Pl.* plating, *R* retrospective, *RC* retrospective comparative, *TFW* transfixing wires

**Table 2:** Follow-up details, and sources of clinical diversity

Sort	Authors	F-up (months) mean (range)	Previous surgeries, mean (range)	Soft tissue cover	AB treatment	EF time, (months): mean (range)	Bone grafting (*n*-cases)
1	Magadum [30]	27 (25–39)	2 (1–4)	PC (due to AS)	nr	10.2 (4.5–24)	nr
2	Baruah [31]	(24–72)	nr	nr	nr	6.1 (3.3–14.6)	0
3	Madhusudhan [32]	13 (6–20), post-union	3 (2–5)	nr	nr	9 (4–13)	0
4	Bumbasirevic [33]	99 (62–122)	1.3 (1–3)	Soft-tissue transport STSG: 4	No use of systematic abs	9.7 (7.2–15)	1
5	Liu [34]	72.5 (35–106)	2.7 (1–6)	Free/local flaps: 5	3 wks IV	nr	2
6	Mora [35]	2–26 years	nr	Epidermo-fascial osteoplasty (Umiarov)	Adequate AB (not specified!)	nr	14
7	Xu [36]	29 (12–72)	6 (3–14)	AS – use of local soft tissue	IV AB for 1 wk p-op	10 (8–14)	nr
8	Moghaddam [17]	12	6.7 (1–31)	nr	2–4 wks: IV, 2–4 wks: orally	na	RIA: 50, Il crest: 13, BMP: 50, Allog. 37
9	Barakat [37]	At 2, 6,12, and 24 months	1.5 (1–4)	AS/PC	Adequate ABs for at least 8 wks	nr	nr
10	Meleppuram [38]	14 (10–24)	nr	nr	Appropriate IV Abs for a week	(8–10)	13
11	Tong [39]	Mq: 23.1 ± 9.5 DO: 27.5 ± 10.1	nr	VAC, Free flaps, STSG in 50% of cases	2–4 wks: IV, 2–4 wks: orally	Mq: 10.1 ± 1.7 DO: 17.2 ± 3.8	Il. crest: 13, BMP: 13 nr
12	Morris [18]	23 (13.4–32)	nr	Free flaps: 3, Local flaps: 5, STSG: 3, PC:1	Not specified	n.a	Il. crest: 12, BMP: 5
13	Testworth [40]	41 (12–84)	4.5	nr	nr	11.3 (4–23)	nr
14	Luo	22.5 (18–35)	2.3	Free or local flaps: 21	× 2 wks after 1st st and × 24 h after 2nd stage	n.a	Il. crest: all 67 cases
15	Siboni [41]	34 (12–82)	3.6	Free flaps: 5, Local flaps: 5, PC: 13	Adapted ab treatment	n.a	Il. crest: 18 Allog: 9
16	Bakhsh [42]	20 (7–36) post frame removal	nr	Not specified	Not specified	9.3 (5–16)	0
17	Rohilla [43]	22 (12–30)	nr	PC with relaxing incisions. STSG: 3	Br-sp. IV Abs × 5 days + oral × 2 wks	8.6 (5–18)	nr
18	Jilani [44]	11.3 (8.3–22)	2.4 (1–5)	Plastic cover: 8 cases (not specified)	IV ABs x 4d Oral Abs till stitch removal	8.2 (7–19)	2
19	Sharma [45]	15 (12–26)	3 (2–5)	nr	As per culture sensitivity	3.8 (3–4.5)	nr
20	Kinik [51]	32.5 (12–72)	2.9 (1–9)	nr	3 wks: IV, 3 wks: oral	13.7 (8–27)	nr
21	Baruah [46]	(24–72)	5	Cross-leg: 2, local flaps: 5, STSG: 9	nr	nr	nr
22	Sigmund [47]	37.9: median (16–128)	2.8 (1–6)	Muscle flaps: 17	Appropriate Abs: at least 6 wks	9.7 (5–17)	8 (5 BG + 3 BMPs)
23	Kushwaha [48]	29.5 (16–50)	nr	nr	nr	11.2 (6–18)	nr
24	Wadhwani [49]	35 (minimum 6 years post frame removal)	nr	STSG:5	2 weeks	10.5	nr
25	Yushan [50]	29.5 (24–38)	2.8	Free flap: 6, Local flap: 4, during BT: 3	6 wks IV	13.8	nr
26	Pesciallo [19]	26 median (13–54)	nr	Local flaps: 5, STSG:3, PC:5	Adapted AB treat	nr	Il. crest: 13 + Allog: 13
27	Lotzien [52]	33 (13–69)	7.9 (1–22)	Free flaps: 19, Local flaps: 4	nr	10.3 (4–21)	RIA: 30, Il crest: 18, BMP: 2, Allog. 23
28	Gupta [53]	> 9 months	nr	Local muscle flaps in some cases (not specified)	Not specified	nr	3
29	Rohilla [54]	DO: 31.6 (24–37) MQ: 30.4 (24–36)	nr	STSG or flaps: 7	DO: 2 wks IV 2 wks: oral MQ: 2 wks IV 2 wks: oral	DO: 9.4 (6–12) MQ: 16.3 (13–18)	nr Il crest: 12
30	Yang [20]	18 (12–32)	1 (0–3)	nr	6 wks after 2nd stage	nr	Il. crest: 12 ± Allog
31	Ozpolat [55]	24.6 (13–40)	2.8 (1–6)	Free flaps: 3, Local flaps: 4	2–4 wks IV, + 2 wks PO	nr	Il. crest: 11 Allog: 10
32	Corona [56]	48 (15–110)	3.4 (0–15)	Free flap: 21, Local flap: 10, VAC: 28	IV: 10d, Oral: 4–6 wks	17.8 (9–32)	11

*AB* antibiotic; *AS* acute shortening, *Allog.* supplementary allograft, *BG* bone graft, *BMP* bone morphogenetic protein, *Br-sp.* broad-spectrum, *DO* distraction osteogenesis, *EF* external fixation, *Mq* masquelet (technique), *nr* not reported, *n.a.* not applicable, *PC* primary closure, *RIA* reamer irrigation aspiration, *STSG* split-thickness skin graft, VAC vacuum-assisted closure

### Assessment of the risk of bias

With regards to LoE, there were 2 RCTs (Level I) [43, 54], 5 prospective cohort studies (Level II) [32, 45, 47, 48, 53] 7 retrospective comparative studies (Level III) [17, 31, 39, 40, 46, 49, 50] and 18 retrospective cohort studies (Level IV) [18–20, 30, 33–38, 41, 42, 44, 51, 52, 55–57] (Table 3). The overall risk of bias of both randomised prospective trials [43, 54] was high, according to the RoB2 tool (Table 3). The MINORS score across all primary studies averaged 11 points (median score = 10), ranging from 7 to 20 points. The wide range of scores was undoubtedly due to the design of each primary study (excluding methodological quality), as the studies containing a comparator group received an additional rating (Table 3).

**Table 3:**
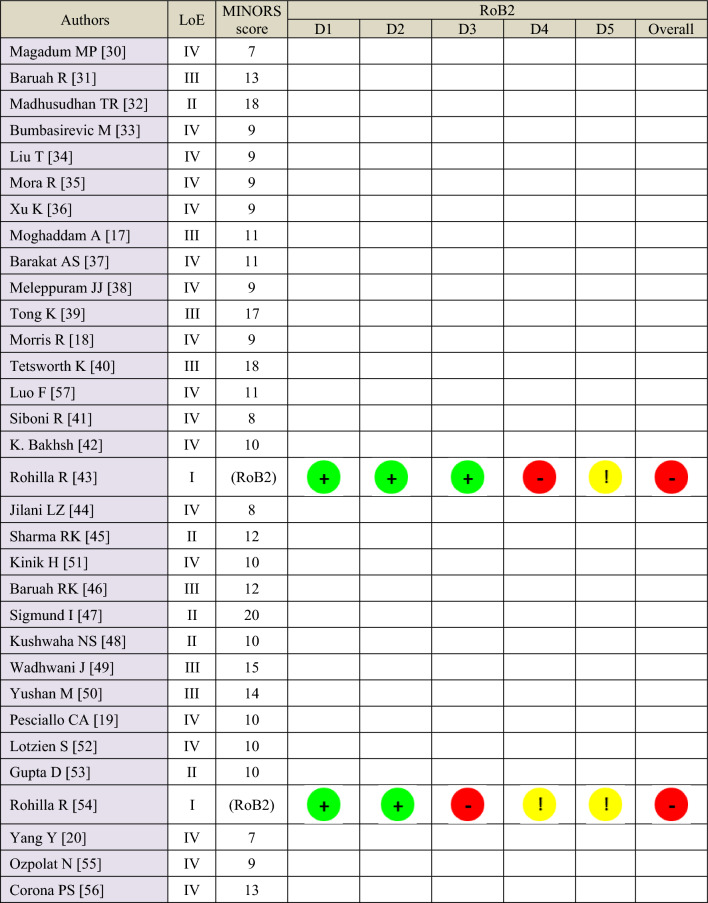
Level of evidence (LoE) and risk of bias assessment of the primary studies

D1: bias due to the randomisation process. D2: bias due to deviations from intended interventions. D3: bias due to missing outcome data. D4: bias in measurement of the outcome. D5: bias in selection of the reported result

Low risk of bias,  Some concerns,  High risk of bias

### Publication bias

**Fig. 2:**
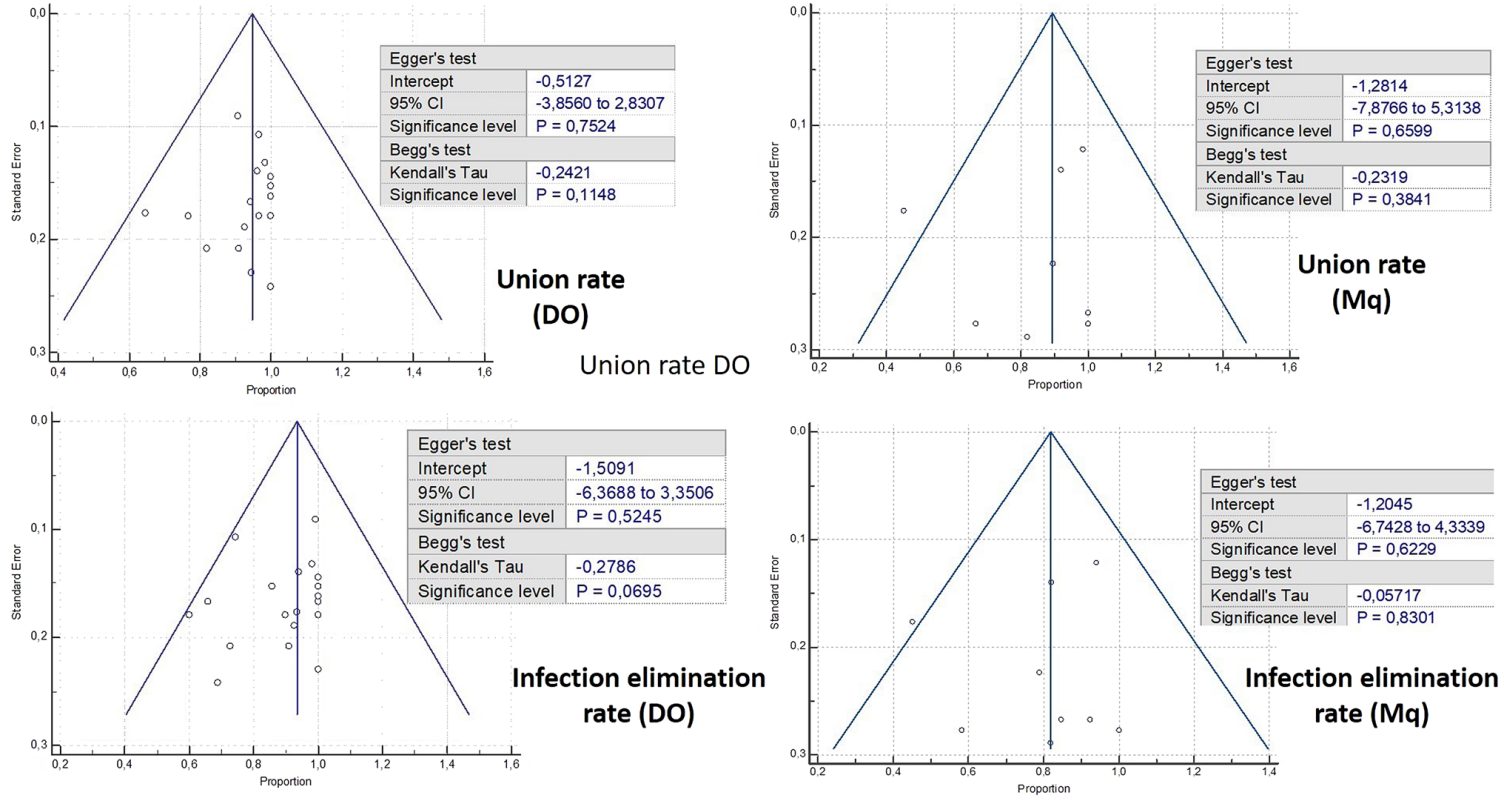
Funnel plots demonstrating union rates and infection elimination rates between the Ilizarov and Masquelet groups

For the meta-analysis of studies directly comparing Mq against Ilizarov, assessment of publication bias was not possible due to the limited number of component studies (*n = *2) [39, 54]. For the single-cohort meta-analyses, we generated respective funnel plots for all primary outcomes of interest. The distribution of data-points was symmetrical across the vertical line corresponding to the pooled effect estimate and within the confines of the inverse funnel plot (Fig. 2). In addition, the calculations of Egger’s test and the Begg’s rank test yielded p-values well-above the significance level, indicating that publication bias was unlikely.

### Meta-analysis of studies with comparator cohorts

Only two studies attempted a direct comparison of Ilizarov against Mq for the management of infected tibial NUs, including 51 patients [39, 54]; one of these studies was a retrospective comparative study reporting on 26 patients (13 patients treated with the DO method and the remaining 13 patients with the Mq technique) [39], while the other study was a RCT including 25 patients (13 of them treated with the DO method and the remaining 12 with the Mq technique) [54]. Using the inverse variance method and a random effects model, no statistically significant difference was documented in the risk ratio for the following outcomes: union, infection elimination, amputation and Association for the Study and Application of the Method of Ilizarov (ASAMI) bone and functional results (Table 4).

**Table 4:** Results of the meta-analysis of the studies with comparator groups

Outcome	Studies [References]	Events/totals		Effect estimate (RR) [95% CI]	Heterogeneity
		Ilizarov	Masquelet		
Union rate	2 [39, 54]	25/26	19/25	1.27 [0.71, 2.28], *p = *0.43	*Q = *3.96, *df * = 1 *p = *0.05, *I*^2^ = 75%
Infection elimination rate	2 [39, 54]	24/26	19/25	1.24 [0.63, 2.41], *p = *0.53	*Q = *4.72, *df* = 1 *p = *0.03, *I*^2^ = 79%
Amputation rate	2 [39, 54]	0/26	2/25	0.19 [0.01, 3.52], *p = *0.26	Not applicable
Excellent/good bone results (ASAMI)	2 [39, 54]	21/26	16/25	1.26 [0.62, 2.54], *p = *0.52	*Q = *3.51, *df * = 1 *p = *0.06, *I*^2^ = 72%
Excellent/good functional results (ASAMI)	2 [39, 54]	21/26	18/25	1.13 [0.74, 1.72], *p = *0.57	*Q = *1.76, *df* = 1 *p = *0.18, *I*^2^ = 43%

*ASAMI* association for the study and application of the method of Ilizarov; *CI* confidence interval; *df* degrees of freedom

### Meta-analysis of single-cohort studies

Studies reporting exclusively on either the Ilizarov or Mq method for the management of septic tibial NUs, as well as single treatment arms in retrospective comparative studies, were processed separately in a proportion meta-analysis. There were 22 studies reporting on the DO method [30–38, 40, 42–51, 53, 56] and 9 on the Mq IMT method [17–20, 39, 41, 52, 55, 57].

### Primary outcomes of interest

I. Union rate

**Fig. 3:**
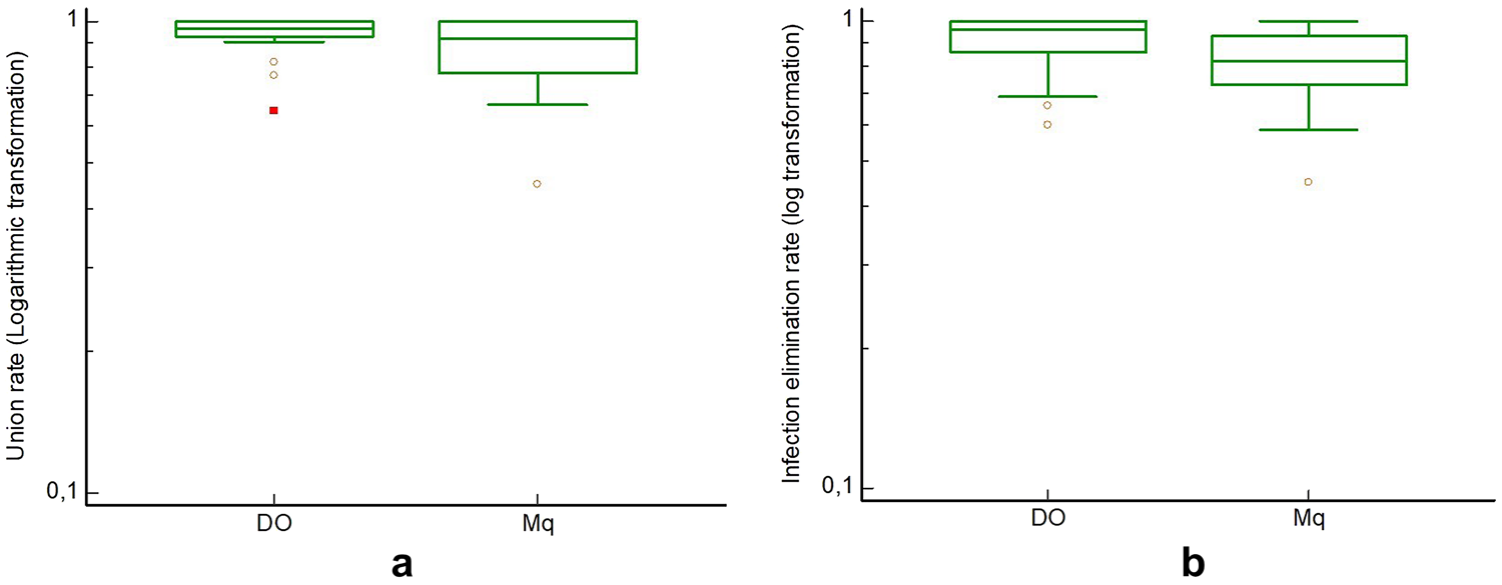
Boxplots of **a** union rate and, **b** the infection elimination rate between the Ilizarov and Masquelet groups

For both the DO and IMT groups, all respective primary studies (22 studies reporting on 865 patients that had been treated with the Ilizarov method, and 9 studies including 228 patients that had been treated with the Mq technique) provided relevant data [17–20, 30–53, 55–57]. The overall effect estimate for the union rate was 94.6%, (95% CI 91.6–97%, *Q = *65, *df*: 21, *I*^2^ = 68%, random effects model) for the Ilizarov group, and 88.0% (95% CI 75–97%, *Q = *53, *df* 8, *I*^2^ = 85%, random effects) for the Mq group. A non-parametric comparison of union rate between the two aforementioned techniques showed no statistically significant difference (*p = *0.45, Mann–Whitney *U* test), (Fig. 3).

II. Infection elimination rate

All primary studies in both treatment groups provided relevant data [17–20, 30–53, 55–57]. The pooled effect estimate of infection elimination rate was 93.0% (95% CI 88–97%, *Q = *140, *df*: 21, *I*^2^ = 85%, random effects) for the Ilizarov treatment group, and 80.4% (95% CI 67.5–90.6% *Q = *38, *df*: 7, *I*^2^ = 79%, random effects) for the Mq group. No statistically significant difference could be established between the two treatment methods in terms of infection elimination rate (*p = *0.06, Mann–Whitney *U* test), (Fig. 3).

### Secondary outcomes of interest in both treatment cohorts

We calculated pooled effect estimates of the following outcomes of interest for both treatment groups: (i) unplanned reoperation rate; (ii) amputation rate; and (iii) refracture rate. No statistically significant difference was documented for all above outcomes of interest between DO and Mq treatment options (Table 5).

**Table 5:** Secondary outcomes of interest for DO and Masquelet treatment groups

Outcome	Treatment group	n-studies (cohort size)	Pooled effect estimate, 95% CI [meta-analysis model]	Heterogeneity	*p* (Mann–Whitney *U* test)
Unplanned re-operation rate	DO	16 (707 pts)	**24%**, 13–36%, [random effects]	*Q = *203, df: 15, *p < *0.0001, *I*^2^ = 93%	0.4
	Masquelet	6 (134 pts)	**32.5%**, 14.5–54%, [random effects]	*Q = *28, df: 5, *p < *0.01, *I*^2^ = 82%	
Amputation rate	DO	18 (698 pts)	**1.3%**, 0.6–2.4%), [fixed-effects]	*Q = *14.4, df: 17, *p = *0.6, *I*^2^ = 0%	0.07
	Masquelet	9 (*2*28 pts)	**5%**, 1.9–9.7%, [random-effects]	*Q = *14, df: 8, *p = *0.08, *I*^2^ = 42.8%	
Re-fracture rate	DO	10 (352 pts)	**4.4%**, 2.5–7%, [fixed-effects]	*Q = *2.7, df: 9, *p = *0.1, *I*^2^ = 0%	0.25
	Masquelet	4 (122 pts)	**3.2%**, 0.9–8%, [fixed-effects]	*Q = *1.8, df: 3, *p = *0.6, *I*^2^ = 0%	

*P*<0.05 was set as the threshold for significance (in bold)

*DO* distraction osteogenesis; *CI* confidence interval; *df* degrees of freedom

### Outcome and complications inherent to the DO method

I. Bone graft rate

Eleven studies (472 patients) provided relevant data on the use of bone graft in the DO method [32–35, 38, 42, 44, 46, 47, 53, 56]. The summarized estimate of effect size of the bone grafting rate (at either the docking site or the regenerate bone) was 10% (95% CI 4–18%, *Q = *64, *df*: 10, *p < *0.0001, *I*^2^ = 84%, random effects).

II. ASAMI Bone Results

Relevant data were obtained from all 22 primary studies of DO treatment group [30–38, 40, 42–51, 53, 56]. Based on the calculated pooled effect estimate, 89% of the patients achieved excellent or good bone results (95% CI 83–94%, *Q = *132, *df*: 21, *p < *0.0001, *I*^2^ = 84%, random effects).

III. ASAMI Functional Results

The pooled effect estimate of excellent or good functional results, obtained from 21 primary studies reporting on a total of 823 patients, was 87% (95% CI 80–92%, *Q = *118, *df*: 20, *p < *0.0001, *I*^2^ = 83%) [30–35, 37, 38, 40, 42–51, 53, 56].

IV. Complications inherent to the Ilizarov DO technique

The most frequently encountered complication was pin-track infection (60%), followed by adjacent joint stiffness (26%), LLD (19%), residual equinus deformity (16%) and delayed consolidation of the regenerate bone (11%) (Table 6).

**Table 6:** Complications within the Ilizarov group

Outcome	*n*-studies (cohort size)	Pooled effect estimate, 95% CI [meta-analysis model]	Heterogeneity
Pin-track infection	20 (763)	**60%**, 44–76%, [random effects]	*Q = *401, *df*: 19, *p < *0.001, *I*^2^ = 95%
LLD	5 (159)	**19%**, 1–53% [random effects]	*Q = *86, *df*: 4, *p < *0.001, *I*^2^ = 95%
Residual angulation	12 (400)	**10%**, 6–15%, [random effects]	*Q = *25, *df*: 11, *p = *0.008, *I*^2^ = 56%
Residual equinus deformity	12 (449)	**16%**, 5–30%, [random effects]	*Q = *161, *df*: 11, *p < *0.0001, *I*^2^ = 93%
Adjacent joint stiffness	15 (530)	**26%**, 14–40%, [random effects]	*Q = *183, *df*: 14, *p < *0.001, *I*^2^ = 92%
Delayed consolidation of the regenerate bone	11 (523)	**11%,** 6.4–16%, [random effects]	*Q = *31, *df*: 10, *p = *0.0006, *I*^2^ = 67%

*P*<0.05 was set as the threshold for significance (in bold)

*LLD* leg-length discrepancy; *CI* confidence interval; *df* degrees of freedom

### Subgroup analysis

Firstly, the primary outcomes of interest in the predefined subgroups of both the DO and Mq IMT treatment cohorts were assessed. Circular frames, compared to the mono-lateral external fixation devices, did not appear to offer any clear advantage in terms of union rate (*p = *0.4). The infection elimination rate was higher when using circular frames compared with mono-lateral frames, however this difference was not statistically significant (*p = *0.36) (Table 7). The Mq technique, utilising internal fixation methods (plates or intramedullary nails (IMN)) for the NU site, demonstrated superior union and infection elimination rates than when using external fixation devices; this difference however did not reach statistical significance (*p = *0.84 for bone union, *p = *1.0 for infection elimination) (Table 7). Furthermore, the results of the subgroup analysis for the secondary outcomes of interest (unplanned re-operations, refractures and amputation rates) are depicted in Table 8. With regards to these outcomes, no statistically significant difference was established within the various subgroups (Table 8).

**Table 7:** Subgroup analysis of the primary outcomes of interest in Ilizarov and Masquelet groups

Outcome	Treatment groups	*n*-studies (cohort size)	Pooled effect estimate, 95% CI [meta-analysis model]	Heterogeneity	*p* (Mann–Whitney test)
Union rate	D.O. (circular frames)	15 (672 pts)	95%, 92–98%, [random effects]	*Q = *49, *df*: 14, *p < *0.0001, *I*^2^ = 72%	0.4
	D.O. (Unilateral frame)	7 (178 pts)	94%, 88.6–97.6%, [random effects]	*Q = *9.7, *df*: 6, *p = *0.13, *I*^2^ = 38%	
	Masquelet (ex-fix)	2 (43 pts)	77.5%, 15–97%, [random effects]	*Q = *17.6, *df*: 1, *p = *0.0001, *I*^2^ = 94%	0.84
	Masquelet (IF)	5 (153 pts)	90.5%, 79–98%, [random effects]	*Q = *14, *df*: 4, *p = *0.007, *I*^2^ = 71.2%	
Infection elimination rate	D.O. (circular frames)	15 (672 pts)	95%, 90–98%, [random effects]	*Q = *76, *df*: 14, *p < *0.0001, *I*^2^ = 82%	0.36
	D.O. (Unilateral frame)	7 (178 pts)	86%, 68–98%, [random effects]	*Q = *55, *df*: 6, *p < *0.0001, *I*^2^ = 89%	
	Masquelet (ex-fix)	2 (43 pts)	77.5%, 15–97%, [random effects]	*Q = *17.7, *df*: 1, *p < *0.001, *I*^2^ = 94%	1.0
	Masquelet (IF)	5 (153 pts)	82%, 70–92%, [random effects]	*Q = *11, *df*: 4, *p = *0.03, *I*^2^ = 63%	

*D.O.* distraction osteogenesis; *IF* internal fixation; *ex-fix* external fixator; *CI* confidence interval; *df* degrees of freedom

**Table 8:** Subgroup analysis of the secondary outcomes of interest in Ilizarov and Masquelet groups

Unplanned re-operation rate	D.O. (circular frames)	12 (585 pts)	26%, 12–42%, [random effects]	*Q = *200, *df*: 11, *p < *0.001, *I*^2^ = 94%	0.4
	D.O. (Unilateral frame)	4 (107 pts)	17%, 10.5–24%, [fixed effects]	*Q = *1.2, *df*: 3, *p = *0.7, *I*^2^ = 0%	
	Masquelet (ex-fix)	Insufficient data			
	Masquelet (IF)	4 (103 pts)	29.8%, 9.4–56%, [random effects]	*Q = *17, *df*: 3, *p = *0.0007, *I*^2^ = 82%	
Refracture rate	D.O. (circular frames)	7 (258 pts)	5%, 2.6–8.2%, [fixed-effects]	*Q = *1.5, *df*: 6, *p = *0.9, *I*^2^ = 0%	0.12
	D.O. (Unilateral frame)	3 (94)	3%, 0.7–9%, [fixed effects]	*Q = *0.6, *df*: 2, *p = *0.7, *I*^2^ = 0%	
	Masquelet (ex-fix)	Insufficient data			
	Masquelet (IF)	3 (91 pts)	2%, 0.25–7.4%, [fixed effects]	*Q = *0.006, *df*: 2, *p = *0.99, *I*^2^ = 0%	
Amputation rate	D.O. (circular frames)	11 (483 pts)	1.2%, 0.5–2.5%, [fixed effects]	*Q = *9.6, *df*: 12, *p = *0.6, *I*^2^ = 0%	0.8
	D.O. (Unilateral frame)	5 (127 pts)	2%, 0.2–5%, [random effects]	*Q = *4.5, *df*: 4, *p = *0.3, *I*^2^ = 11%	
	Masquelet (ex-fix)	2(43 pts)	7%, 0.7–19%, [random effects]	*Q = *1.4, *df*: 1, *p = *0.23, *I*^2^ = 29%	1.0
	Masquelet (IF)	5 (153 pts)	5%, 0.7–13%, [random effects]	*Q = *10.7, *df*: 4, *p = *0.03, *I*^2^ = 63%	

*D.O.* distraction osteogenesis; *IF* internal fixation; *ex-fix* external fixator; *CI* confidence interval; *df* degrees of freedom

### Sensitivity analysis

We repeated the pooled analysis after excluding low-rating studies according to the MINORS instrument (score < 10) [20, 30, 33–36, 38, 41, 44, 55] (Table 9). The obtained results did not differ from the original figures obtained and, thus, we feel confident that our findings are robust.

**Table 9:** Sensitivity analysis

	*n*-studies [references]	*n*-patients	Pooled effect estimate (95% CI)	Heterogeneity
DO				
Union rate	15 [31, 32, 37, 40, 42, 43, 45–51, 53, 56]	560	94.5%* (90–97.6%), random effects	*Q = *53, *df * = 14, *p < *0.01, *I*^2^ = 74%
Infection elimination rate	15 [31, 32, 37, 40, 42, 43, 45–51, 53, 56]	560	89.7%^@^ (82–95.5%), random effects	*Q = *102, *df* = 14, *p < *0.01, *I*^2^ = 86%
Amputation rate	11 [31, 32, 40, 42, 43, 45, 47, 50, 51, 53, 56]	393	1.5%^&^ (0.6–3%), fixed effects	*Q = *5.5, *df* = 10, *p = *0.85, *I*^2^ = 0%
Mq				
Union rate	5 [17, 19, 39, 52, 57]	174	90.4%* (70–99.7%), random effects	*Q = *46, *df * = 4, *p < *0.01, *I*^2^ = 91%
Infection elimination rate	5 [17, 19, 39, 52, 57]	174	80.2%^@^ (61–94%), random effects	*Q = *29, *df * = 4, *p < *0.01, *I*^2^ = 86%
Amputation rate	5 [17, 19, 39, 52, 57]	174	3.5%^&^ (0.5–9%), random effects	*Q = *8.7, *df * = 4, *p = *0.07, *I*^2^ = 54%

*DO* distraction osteogenesis; *Mq* masquelet; *df* degrees of freedom

*p = 0.96, ^@^*p = *0.31, ^&^*p = *0.81, Mann–Whitney *U* test

### Quality of evidence

Based on the transparent framework of the GRADE tool, the overall quality of evidence of the primary outcomes of interest, and the ASAMI bone and functional results was low (Table 10). This could be attributed to the fact that they were based on a large number of studies with a lower LoE and, therefore, were prone to several sources of bias.

**Table 10:** GRADE quality assessment of the primary outcomes of interest and ASAMI bone and functional results

Outcome of interest	*n*-studies	LoE: *n*-studies	GRADE analysis								
			Risk of Bias	Inconsistency	Indirectness	Imprecision	Publication bias	Large effect	Dose effect	Biases possibly reducing treatment effect	Overall GRADE quality
Union rate	32	I: 2, II: 5, III: 7, IV: 18	X	V	V	X	V	V	na	X	(+ +)
Infection elimination rate	32	I: 2, II: 5, III: 7, IV: 18	X	X	V	X	V	V	na	X	(+ +)
ASAMI bone (excellent-good)	24	I: 2, II: 5, III: 5, IV: 12	X	X	V	X	V	V	na	X	(+ +)
ASAMI functional (excellent-good)	23	I: 2, II: 5, III: 5, IV: 11	X	X	V	X	V	V	na	X	(+ +)

GRADE Factors: V: no serious limitations; X: serious limitations; n.a.: not applicable. Overall quality of evidence: 0: very low; + : low; +  + : moderate; +  +  + : high

*GRADE* Grading of Recommendations Assessment, Development and Evaluation; *ASAMI* Association for the Study and Application of the Method of Ilizarov; *na* not applicable

## Discussion

This systematic review highlighted that the outcomes of both direct and indirect comparisons between DO and Mq IMT methods in management of infected tibial NUs with concomitant osseous defects, was unequivocal.

The Mq IMT method has been associated with variable outcomes in the literature. A previous systematic review, comprising of 17 primary studies, reported union and infection elimination rates ranging from 67 to 100% [58]. The pooled effect estimates of our study for both union rate (88%, 95% CI 75–97%), and infection elimination rate (80.4%, 95% CI 67.5–90.6%)) are in line with the results of the previous systematic review [58]. It should be emphasised, however, that our study focused exclusively on infected tibial NUs with concomitant segmental bone loss, while the former systematic review included cases with bone defects of various aetiology (including acute trauma bone loss, infected and aseptic NUs, and bone defects following tumour resection); and anatomical location (tibia, femur, fibula, humerus, and forearm bones).

Various clinical series have also reported on the outcomes of the Mq technique. Masquelet et al. were the first to report on the complications of their own technique: infected NU rate at 14.3% in a series of 35 patients [11]. Since then, the reported union rate of the Mq technique has been markedly variable, ranging from as low as 42% [18] to as high as 98% [59]. This great discrepancy in reported outcomes may be attributed to the diversity of the included patient cohorts within the various clinical series, in terms of the anatomical location of the osseous defect, the condition of the surrounding soft tissue envelope and the initial presence of infection. As for the latter, it has been shown that the presence of infection at the commencement of the Mq technique constitutes a risk factor for failure [60]. Residual surgical site infection has been established as the main cause of failure of the Mq technique and, therefore, a thorough and meticulous surgical debridement is recommended during both stages of the Mq procedure [13]. As a result, the number of procedural stages may be more than two depending on infection status and the requirement of infection elimination for bone graft insertion [3, 12]. Multiple debridements must also be repeated during the final stage of spacer replacement by bone graft and definitive fixation of the NU, as this stage presents the final opportunity to remove avascular and thus potentially contaminated bone [3, 12, 13]. Failure to understand the value of achieving a local sterile environment before embarking on the final stage of definitive bone grafting and stabilisation of the defect site is perhaps, the main reason for producing suboptimal outcomes with the IMT in several clinical series.

The adequacy of osseous debridement in the IMT, which is intimately associated with the generated bone defect, raises concerns regarding the healing capacity of large osseous defects and the amount of autologous bone graft required to fill the defect site. However, it has been demonstrated that the healing time in the IMT is independent of the size of the osseous defect [61]. In addition, the development of autologous bone graft harvesting techniques, together with the evolution in the production of volume-expanding materials, have adequately addressed the issue of excessive graft requirements [14–16].

The osteosynthesis construct at the final stage of the Mq method seems to influence the ultimate outcome by affecting the mechanical environment at the NU site. An adequately stable mechanical environment, produced by either IMNs or extramedullary devices (plates/screws) of optimal working length is of paramount importance for the successful healing of the grafted osseous defect [12]. It has been argued that external fixation devices (either mono-lateral or circular frames) are more prone to loosening, resulting in suboptimal mechanical conditions to promote healing of the bone defect [12]. In the subgroup analysis comparing the outcomes of the IMT technique in different mechanical environments (external versus internal fixation devices), although improved union and infection elimination rates were achieved with the use of internal over external fixation devices, the established differences did not reach statistical significance; this was likely due to the small cohort size (Table 7).

Ilizarov DO is presently one of the most effective methods of managing large bone defects, particularly when compounded by infection. Its efficacy lies with the fact that it allows for a generous bone debridement and removal of all avascular and potentially contaminated bone with subsequent gradual reconstruction of the generated large osseous defect. Its obvious advantage over the Mq method is that it does not require large amounts of autologous bone graft to fill the osseous defect. However, in contrast to the IMT, the ultimate success of the DO depends on the defect size, as large bone gaps require prolonged external fixation time, which in turn, increases complication rates and raises issues of patient compliance and tolerance [2].

The most frequently documented complications of the DO method in our study were pin-track infection, recorded in as high as 60% of cases, followed by adjacent joint stiffness (particularly in the form of residual equinus deformity) and LLD (Table 6). Pin-track infections are related to the external fixation time and, indirectly, to the size of the initial bone defect [62]. Adjacent joint stiffness arises as a result of muscles contractures that fail to adjust to the increase of bone length [63]. Although it has been demonstrated that an increase in the muscle length and, thus, the avoidance of muscle contractures, could be promoted by early weight bearing; it does not however reach the levels of the respective osseous elongation [64]. Therefore, in cases of large osseous defects with increased length requirements of the regenerate bone, muscle contractures are inevitable and should be considered as a noteworthy disadvantage of this Ilizarov DO method.

The anatomical site of the joint stiffness is associated with the direction of bone transport. A large proximal-to-distal bone transport could induce contractures of the gastrocnemius and knee stiffness, while a distal-to-proximal bone transport, by transferring the tibialis posterior and flexor digitorum longus muscles proximally, would result in equinus and toe flexion-contracture deformities [63]. The increased frequency of LLD with the DO method warrants further research exploration. This could be attributed to a technical error, loss of reduction, frame loosening or muscle contracture, which may induce premature bone consolidation and inhibit further bone lengthening [62]. The use of mono-lateral frames in infected tibial NUs did not seem to demonstrate a clear benefit over the circular frames used in the classical Ilizarov method, with regards to of bone union (Table 7). However, the infection elimination rate was significantly lower with the use of mono-lateral frames when compared with the circular ones. This finding might be explained by the fact that the classical Ilizarov circular frames provide a much more stable mechanical environment and, thus, allow the surgeon to perform a more radical osseous debridement than the mono-lateral frames.

Other advantages of the Ilizarov circular frames include their ability to be adjusted during DO, to correct ensuing deformities. They also permit early weight-bearing. On the other hand, the pain produced by the tensioned wires of the circular frames in the tibial region is tolerable and does not constitute a significant drawback compared to the mono-lateral frames. Perhaps, the mono-lateral frames are more appropriate for application in the femur region. This may be attributed to the bulky soft tissue envelop and the presence of neurovascular structures within the three out of its four compartments which make the use of tensioned wires challenging in the tibial region, and therefore provoke significant pain which is not always tolerated by the patient [65].

Despite a rigorous search of the existing literature, we were only able to identify two relevant primary studies directly comparing DO and IMT for the treatment of infected tibial NUs. The results of direct comparison of the two primary studies with rather small sample sizes [39, 54] suffer from imprecise effect estimates and decreased statistical power. We further identified a number of single-arm, observational studies, reporting on either the use of IMT or DO for the management of septic tibial NUs, and attempted to make an indirect comparison between the two techniques, as relevant, high quality clinical trials were not available. Although the latter constitutes the best evidence for guiding clinical practice when such high-quality evidence is not available, synthesis of data derived from lower evidence-levelled studies remain the cornerstone of clinical practice [66, 67]. Systematic reviews based on non-randomised, observational studies lack internal validity and are prone to many sources of bias [68]. Our research aimed to limit bias by adhering to a succinct and structured study protocol and registered prospectively to the PROSPERO database prior to the inception of the search strategy. To eliminate heterogeneity across our dataset, we defined a clear and focused research question and subsequently imposed strict eligibility criteria following the PICOS format. We further explored the potential presence of heterogeneity across the recruited material by appropriately designed and performed sub-group and sensitivity analyses based on hypotheses that were generated a priori at the inception of the study protocol. The possibility of having missed sizeable studies that could potentially affect our pooled effect estimates (publication bias) is quite unlikely, as indicated by the generation of appropriate funnel plots and the calculation of Egger’s test and the Begg’s rank test for all primary outcomes of interest. Finally, the quality of the summarised evidence obtained by the meta-analysis was rated based on the transparent framework of the GRADE tool.

It should be noted that there was a general paucity of reported data on Patient Reported Outcome Measures (PROMs) and Quality of Life (QoL) which prevented the gathering of pooled data for comparison of different treatment groups. Consequently, we were unable to offer statistically substantiated comparative effect estimates between the study groups themselves. Moving forwards, studies reporting PROMS and QoL in these settings should be considered and form an important future research agenda.

DO and IMT currently constitute the foundations of modern management of large osseous defects compounded by infection in the tibial region. Despite the fact that the outcomes of both methods appear equivalent in the herein study, our researchers have identified that further refinements in the execution of the IMT, with particular attention to meticulous debridement of all foci of infection through multiple stages before the final stage of bone grafting, could further improve its outcomes in the adverse environment of septic tibial NUs with concomitant bone defects [59].

## Data Availability

The datasets generated and analysed during the current are available from the corresponding author on reasonable request.
